# Effectiveness of noninvasive ventilation for preoxygenation in emergency intubation: a systematic review and meta-analysis

**DOI:** 10.62675/2965-2774.20260128

**Published:** 2026-01-14

**Authors:** Luciana Gioli-Pereira, Victor A Gomez Galeano, Rafael Hortencio Melo, Camila Campos Grisa Padovese, Edielle Sant’Anna Melo, Ary Serpa

**Affiliations:** 1 Hospital Israelita Albert Einstein São Paulo SP Brazil Hospital Israelita Albert Einstein - São Paulo (SP), Brazil.; 2 Hospital Auxilio Mutuo San Pablo Bayamón Puerto Rico Hospital Auxilio Mutuo San Pablo - Bayamón, Puerto Rico.; 3 Universidade Estadual de Londrina Londrina PR Brazil Universidade Estadual de Londrina - Londrina (PR), Brazil.

**Keywords:** Intubation, Noninvasive ventilation, Hypoxia, Oxygenation, Emergency

## Abstract

**Objective:**

To evaluate the impact of noninvasive ventilation *versus* bag-valve-mask ventilation preoxygenation on safety and efficacy outcomes.

**Methods:**

PubMed, Embase, and Cochrane databases were searched for randomized controlled trials that compared preoxygenation using noninvasive ventilation and bag-valve-mask ventilation. The reported outcomes were all-cause mortality; hypoxemia during intubation; and regurgitation. We perform frequentist and Bayesian analysis. Heterogeneity was examined with I^2^ statistics. Statistical analysis was done using RStudio and Review Manager.

**Results:**

We included 3 randomized controlled trials with 1,555 patients, of whom 771 (49.6%) received preoxygenation with noninvasive ventilation (intervention group). Hypoxemia during intubation was significantly lower in the noninvasive ventilation compared to the bag-valve-mask ventilation group in frequentist with a pooled log RR of −0.94 (log RR −0.94; 95%CI −1.61 − −0.28) and Bayesian pooled log risk ratio of −0.32 (log RR −0.32; 95% credible interval −0.91 − 0.40). Regurgitation was a safety outcome that did not present a difference between the groups in the frequentist or Bayesian models, with log RR −0.50 (log RR −0.50; 95% credible interval −2.09 − 1.01). There was no significant difference between groups in all-cause mortality and other secondary outcomes.

**Conclusion:**

Preoxygenation with noninvasive ventilation significantly reduces the risk of hypoxemia during emergency intubation compared to bag-valve-mask ventilation. However, there were no significant differences in all-cause mortality or regurgitation rates.

## INTRODUCTION

Hypoxemia is a significant concern during tracheal intubation of critically ill patients, as it can lead to severe complications such as cardiac arrest and death. Preoxygenation is a critical step in the intubation process to increase oxygen reserves and reduce the risk of hypoxemia.^(1)^ Various methods of preoxygenation, including noninvasive ventilation (NIV) and bag-valve-mask ventilation (BVMV), are employed to optimize patient oxygenation before intubation. The choice of preoxygenation technique can significantly impact the incidence of hypoxemia and other adverse events during the procedure.^(2)^

Noninvasive ventilation involves the use of a mask or similar device to deliver positive pressure ventilation without the need for an invasive airway. Noninvasive ventilation provides continuous positive airway pressure (CPAP) or bilevel positive airway pressure (BiPAP), which helps maintain alveolar recruitment and improve oxygenation. In contrast, BVMV is a manual method in which a self-inflating bag is used to deliver breath to the patient. Bag-valve-mask ventilation is often used in emergency settings due to its simplicity and availability, but it requires skilled operators to ensure adequate ventilation and avoid complications such as gastric insufflation.^(3,4)^

Recent studies have demonstrated that NIV is more effective than BVMV in reducing the incidence of hypoxemia during intubation. For instance, a multicenter randomized trial found that preoxygenation with NIV resulted in a significantly lower incidence of hypoxemia compared to an oxygen mask, with hypoxemia occurring in 9.1% of patients in the NIV group *versus* 18.5% in the oxygen mask group.^(1)^ Additionally, a network meta-analysis indicated that NIV is superior to conventional oxygen therapy and high-flow nasal cannula in maintaining higher SpO_2_ levels during intubation.^(2)^

Given the critical importance of optimizing preoxygenation to prevent life-threatening hypoxemia during intubation, this meta-analysis aimed to evaluate the impact of NIV *versus* BVMV preoxygenation on safety and efficacy outcomes.

## METHODS

### Study design

This systematic review and meta-analysis were performed and reported under the Cochrane Collaboration Handbook for Systematic Reviews of Interventions and the Preferred Reporting Items for Systematic Reviews and Meta-Analysis (PRISMA) statement guidelines.^(5,6)^ The protocol was published in the International Prospective Register of Systematic Reviews, PROSPERO (CRD42024621698).

### Data source and search strategy

We systematically searched PubMed/MEDLINE, Embase, and the Cochrane Central Register of Controlled Trials (CENTRAL) from inception through the final search date of August 1, 2024. In addition, we employed backward snowballing (i.e., reviewing reference lists and related articles) to identify additional relevant studies from those retrieved in the original search. An updated search was performed on April 3, 2025, which yielded no new eligible studies or changed the set of included articles. Three authors performed the systematic review independently, and disagreements were resolved in a panel discussion between authors. Study selection involved screening titles and abstracts followed by a full-text evaluation of potentially eligible studies. The complete search strategy for each database is available in table 1S (Supplementary Material).

### Eligibility criteria

There was no restriction regarding the publication date, status, or language. We considered studies eligible if they were randomized controlled trials (RCTs); enrolled critically ill patients undergoing tracheal intubation; compared preoxygenation with NIV *versus* BVMV, and with no restrictions of follow-up time. In addition, studies were included only if they reported any of the clinical outcomes of interest. We excluded studies with no control group; overlapping study population; without reports on any of the outcomes of interest; and pediatrics, animal studies, reviews, guidelines, and abstracts from congresses.

### Endpoints

Our primary efficacy endpoint was hypoxemia during intubation. Secondary endpoints included all-cause mortality, ventilation time, and intensive care unit (ICU) length of stay (LOS). Our primary safety endpoint was regurgitation. Detailed endpoint definitions for each included study are provided in table 2S (Supplementary Material).

### Quality assessment

Two review authors independently assessed the risk of bias for each trial using the criteria outlined in the *Cochrane Handbook for Systematic Reviews of Interventions*, through Cochrane's Risk of Bias 2 (RoB 2) tool for randomized studies according to the following domains: random sequence generation, allocation concealment, blinding of participants and personnel, blinding of outcome assessment, incomplete outcome data, selective outcome reporting, and other biases.^(7,8)^ We resolved disagreements by discussion or by a third review author. We graded each trial as having a high, low, or unclear risk of bias for each domain.

### Statistical analysis

We conducted both frequentist and Bayesian meta-analyses. For the frequentist analysis, we used a random-effects model based on the DerSimonian and Laird method to pool results for hypoxemia, all-cause mortality, and regurgitation endpoints, reporting risk ratios (RRs) or standardized mean differences (SMDs) with 95% confidence intervals (95%CIs). Heterogeneity was assessed using the I^2^ statistic, and continuous outcomes were analyzed using effect size models.

Additionally, we performed a Bayesian random-effects meta-analysis using the Markov chain Monte Carlo (MCMC) algorithm to estimate pooled effects, reported as posterior means with 95% credible intervals. This approach allowed a probabilistic interpretation of the findings and facilitated the incorporation of prior distributions. Statistical tests for asymmetry (e.g., Egger's or Begg's tests) were not performed due to the limited number of studies.

All statistical analyses were conducted using Review Manager 5.3 (Cochrane Centre, The Cochrane Collaboration, Denmark) and R version 4.3.2 (R Foundation for Statistical Computing, Vienna, Austria), including the *brms* and *metafor* packages.

## RESULTS

### Study selection and baseline characteristics

**Figure 1:**
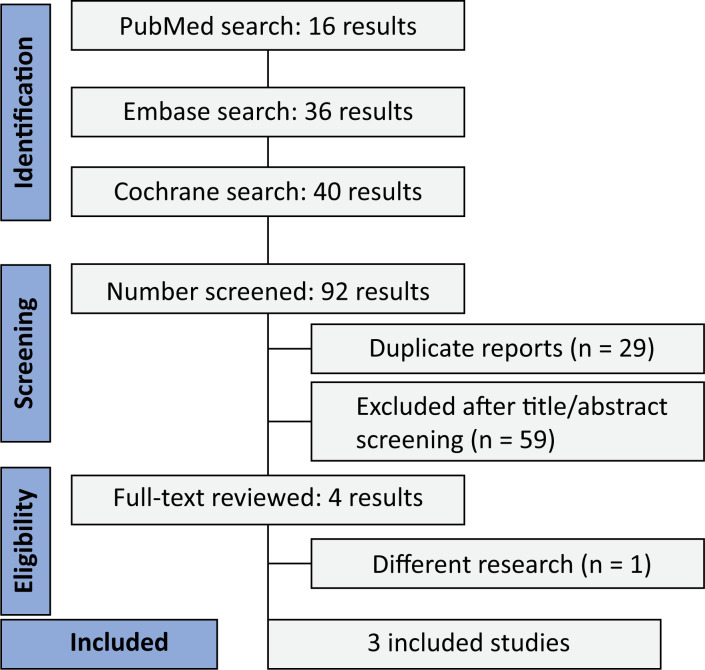
PRISMA flow diagram of study screening and selection.

The study selection is demonstrated in figure 1. The initial search identified 92 studies (PubMed [n = 16], Embase [n = 36], and Cochrane [n = 40]). After title and abstract screening and removal of duplicates, four studies remained to be thoroughly reviewed according to inclusion and exclusion criteria. Of these, 3 RCTs were included,^(1,3,9)^ comprising 1,555 patients, of whom 771 (49.6%) received preoxygenation with NIV (intervention group). The included participants had a mean age of 61.2 years and were predominantly male (61.5%). Study characteristics are in table 1.

**Table 1:** Baseline characteristics of the included studies

Sites	Baillard et al.^(3)^		Baillard et al.^(9)^		Gibbs et al.^(1)^	
	2		6		24	
Group	NIV	BVMV	NIV	BVMV	NIV	BVMV
Patients	27	26	99	102	645	656
Female	10 (37)	7 (27)	33 (33)	33 (32)	255 (39)	260 (40)
Age (years)	64 ± 11	60 ± 15	65 ± 13	63 ± 16	60 ± 18	60 ± 17
BMI (kg/m^2^)	25 ± 7.8	25 ± 5.6	24 ± 3.8	26 ± 6	28 ± 7.1	27 ± 7.4
SAPS II	49 ± 14	51 ± 15	48.7 ± 16.6	47±15.8	NA	NA
APACHE II	NA	NA	NA	NA	17.4 ± 8.2	17.4 ± 8.2
SOFA	NA	NA	6 [4 - 9]	7 [5 - 9]	NA	NA

NIV - noninvasive ventilation; BVMV - bag-valve mask ventilation; BMI - body mass index; SAPS II - Simplified Acute Physiology Score II; APACHE II - Acute Physiology and Chronic Health Evaluation II; SOFA - Sequential Organ Failure Assessment. Results expressed as n, n (%), mean ± standard deviation or median (interquartile range).

### Primary endpoint

**Figure 2:**
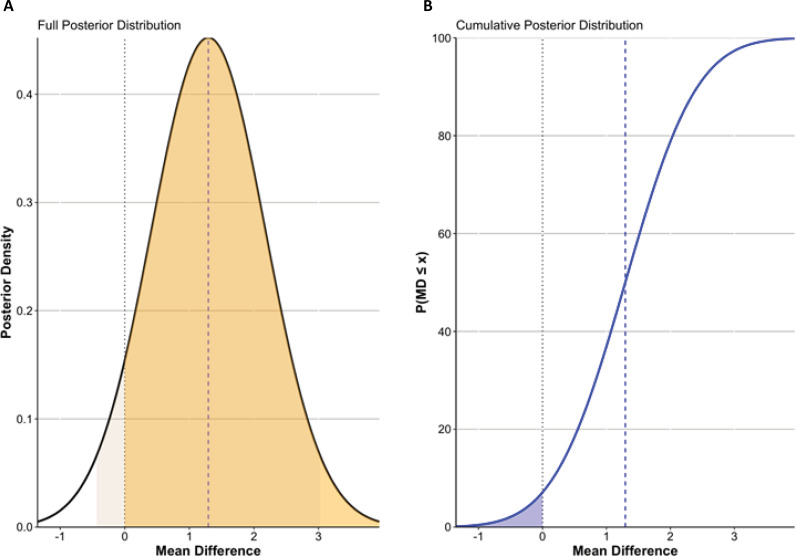
Bayesian analysis of hypoxemia during intubation.

Our analysis demonstrated a reduction in the risk of hypoxemia with NIV compared to BVMV with a Bayesian pooled log RR of −0.32 (log RR −0.32; 95% credible interval −0.91 - 0.40) (Figure 2A). The cumulative posterior probability indicated a high likelihood that NIV was beneficial, with the probability of log RR ≤ 0 exceeding 95%, confirming that the effect likely favored NIV despite some uncertainty (Figure 2B). The frequentist framework similarly favored NIV, with a pooled log RR of −0.94 (log RR −0.94; 95%CI −1.61 - −0.28; p = 0.005) (Figure 1S - Supplementary Material). Individual study estimates showed similar trends: Baillard et al.^(3)^ reported log RR −1.83 (95%CI -3.15 - −0.51; p = 0.007), Baillard et al.^(9)^ log RR −0.65 (95%CI -2.97 - 1.01, p = 0.44), and Gibbs et al.^(1)^ log RR −0.73 (95%CI −1.10 - −0.37; p < 0.001). Both Bayesian and frequentist approaches highlighted a strong likelihood of benefit with NIV, as demonstrated by the Cumulative Distribution Function plot in figure 2B, which shows that nearly all posterior probability distributions lie below zero, reinforcing NIV's superiority in reducing hypoxemia. Table 2 illustrates the probability benefit in each outcome, among all hypoxemias had the highest benefit with about 93%.

(A) Posterior distribution of the Bayesian model showing the estimated mean difference in the risk of hypoxemia between noninvasive ventilation and bag-valve mask ventilation; (B) cumulative distribution function representing the posterior probability that the mean difference exceeds specific thresholds.

**Table 2:** Benefit probability

Outcome	Log RR or mean difference	95% credible interval	Probability of benefit (%)
Hypoxemia	-1	-2.37 - 0.32	~93
ICU length of stay	1.07	-1.15 - 3.27	~38
Mortality	-0.16	-0.82 - 0.47	~58
Regurgitation risk	-0.47	-1.95 - 1.26	~66
Ventilation time	1.31	-1.07 - 3.73	~40

RR- risk ratio; ICU - intensive care unit.

### Secondary endpoints

**Figure 3:**
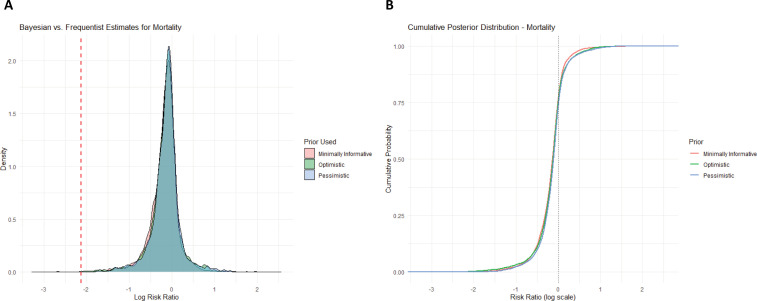
Bayesian analysis of all-cause mortality.

All-cause mortality outcome demonstrated minimal effects, with the Bayesian analysis yielding a pooled log RR of −0.09 (log RR −0.09; 95% credible interval −0.47 - 0.26) (Figure 3A). The cumulative posterior distribution suggests a high probability that the true effect is near zero, reinforcing the absence of a strong mortality benefit (Figure 3B). The frequentist model also showed nonsignificant effects (log RR of −0.07; 95%CI −0.27 - 0.14; p = 0.53), with individual study estimates from Baillard et al.^(3)^ (log RR −0.54; 95%CI −1.20 - 0.15), Baillard et al.^(9)^ (log RR −0.18; 95% CI −0.56 - 0.19), and Gibbs et al.^(1)^ 2024 (log RR 0.00; 95% CI −0.14 - 0.14) (Figure 2S - Supplementary material). Both Bayesian and frequentist approaches confirm a low probability of a meaningful mortality reduction.

(A) Depicts the Bayesian model with priors; (B) the cumulative posterior probability with priors. The red dotted line represents the frequentist point estimate of log (risk ratio).

**Figure 4:**
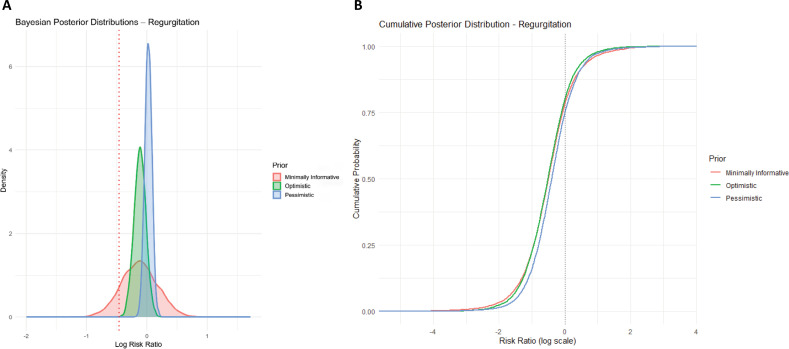
Bayesian analysis of regurgitation.

Given the distribution characteristics of ventilation time and ICU LOS, which deviate from normality, a Bayesian model was used to account for the asymmetry (Figures 3S and 4S - Supplementary Material). The results revealed no statistically significant differences between NIV and BVMV for ventilation time (mean difference [MD] 0.02; 95% credible interval −0.32 - 0.35) and ICU LOS (MD 0.13; 95% credible interval −0.53 - 0.78). The cumulative posterior distributions exhibit substantial overlap. This suggests no clear benefit or harm of NIV for these outcomes.

### Safety endpoint

Regurgitation rates in NIV *versus* BVMV preoxygenation showed no significant differences in either model. The Bayesian analysis yielded a pooled log RR of −0.50 (log RR −0.50; 95% credible interval -2.09 - 1.01), with the cumulative posterior probability suggesting high uncertainty and with no significant association in the frequentist approach, as shown in figure 4. Individual study results were consistent across both methodologies, with Baillard et al.^(3)^ reporting log RR 0.72 (95%CI -2.37 - 1.06), Baillard et al.^(9)^ log RR −0.65 (95%CI -2.49 - 1.15), and Gibbs et al.^(1)^ log RR −0.38 (95%CI −1.19 - 0.34). The cumulative distribution function further illustrates the overlap in posterior distributions, reinforcing the conclusion that NIV does not increase the risk of regurgitation.

(A) Depicts the Bayesian model with priors; (B) the cumulative posterior probability with priors. The red dotted line represents the Frequentist point estimate of log (risk ratio).

### Quality assessment

Individual RCT appraisal using the RoB 2 tool is depicted in figure 5S (Supplementary Material). All studies were deemed as low risk of bias.

## DISCUSSION

We conducted a systematic review and meta-analysis of three randomized controlled trials including 1,555 patients undergoing tracheal intubation, comparing preoxygenation with NIV *versus* BVMV. Our primary analysis demonstrated a reduction in the risk of hypoxemia with NIV compared to BVMV, while no significant differences were observed in all-cause mortality. Additionally, there were no notable differences between the groups in regurgitation rates, ventilation time, or ICU LOS.

The evidence comparing NIV and BVMV for preoxygenation during orotracheal intubation remains limited, particularly concerning safety outcomes such as regurgitation and aspiration. While Baillard et al.^(3,9)^ demonstrated the superiority of NIV in reducing oxygen desaturation in hypoxemic patients, these studies did not comprehensively address regurgitation or aspiration risk. Similarly, Gibbs et al.^(1)^ reported reduced hypoxemia with NIV compared to oxygen mask use, but no statistically significant difference in aspiration rates. Casey et al.^(10)^ also found no significant difference in aspiration rates between BVM and alternative techniques during intubation. These findings underscore the need for future trials with adequate power to evaluate rare but clinically important safety events.

In our study, we conducted a frequentist and Bayesian analysis of the data. The combination of these approaches provides a more comprehensive view of the data, highlighting the robustness of the frequentist findings and the need to consider uncertainty and prior evidence in the interpretation of the results. The American College of Physicians recognizes high-flow nasal oxygen as effective in reducing intubation rates and improving outcomes in acute respiratory failure, while the Surviving Sepsis Campaign highlights the benefits of NIV in improving gas exchange and reducing the work of breathing, noting risks like delayed intubation and aspiration.^(11,12)^ A meta-analysis by Cabrini et al. supports NIV use in acute care, showing reduced mortality and improved outcomes, aligning with our findings of significantly reduced hypoxemia risk with NIV.^(13)^ Guidelines from the European Respiratory Society and American Thoracic Society recommend NIV for conditions like chronic obstructive pulmonary disease (COPD) exacerbations and cardiogenic pulmonary edema. Wedzicha et al. and Yeung et al. further emphasize its role in weaning from invasive ventilation, reducing hospital mortality and ICU stays.^(14,15)^

The Bayesian pooled log RR of −0.32 (95% credible interval −0.91 - 0.40) and nonsignificant results in the frequentist approach suggest no substantial impact of NIV on all-cause mortality. This aligns with the findings of Goury et al., who reported no significant 90-day mortality differences but noted shorter ICU stays with NIV.^(16)^ Similarly, Pettenuzzo et al. found that while NIV reduced re-intubation rates and ICU length of stay in post-operative patients, it did not significantly impact overall hospital mortality.^(17)^ However, Yeung et al. reported reduced mortality with NIV in patients weaned from mechanical ventilation, particularly in COPD patients.^(15)^ This suggests that while NIV may offer benefits in specific contexts, such as reducing re-intubation rates and ICU length of stay, its impact on all-cause mortality remains limited.

The comparison of regurgitation rates between NIV and BVMV preoxygenation shows no statistically significant difference. The frequentist analysis reported a log RR of −0.46 (95%CI: −1.35 - 0.42), and the Bayesian analysis yielded a log RR of −0.50 (95% credible interval -2.09 - 1.01), both indicating no significant difference. However, the wide confidence and credible intervals highlight substantial uncertainty in the pooled effect. Individual study estimates further reflect this variability: Baillard et al.^(3)^ reported log RR 0.72 (95%CI -2.37 - 1.06), Baillard et al.^(9)^ log RR −0.65 (95%CI -2.49 - 1.15), and Gibbs et al.^(1)^ log RR −0.38 (95%CI −1.19 - 0.34). While meta-analysis provides a summary estimate of effect, the wide variability in individual study estimates and the broad CIs and credible intervals indicate considerable uncertainty regarding the magnitude of the effect. Therefore, the results should be interpreted with caution, and further studies are needed to validate these findings.

The findings of our study have significant implications for clinical practice, particularly in the management of patients requiring preoxygenation before orotracheal intubation. The evidence suggests that NIV is effective in reducing the risk of hypoxemia compared to BVMV. This aligns with the guidelines from the American College of Chest Physicians and the American Thoracic Society, which recommend the use of NIV in specific clinical scenarios to improve patient outcomes and reduce the need for reintubation and ICU mortality.^(18)^ In terms of decision-making, the results support the integration of NIV as a standard practice for preoxygenation in high-risk patients. This could lead to updates in clinical guidelines and protocols, ensuring that healthcare providers are equipped with the best practices to manage acute respiratory failure effectively.

These findings indicate that NIV significantly reduces the risk of hypoxemia, with a high posterior probability supporting its benefit. However, no significant impact was observed on mortality, ventilation duration, or ICU length of stay. Bayesian analysis effectively quantified uncertainty, while frequentist models provided complementary insights through CI. Our results highlight the clinical relevance of integrating NIV into preoxygenation strategies for patients requiring intubation. Addressing existing knowledge gaps through further research will enhance patient outcomes and refine clinical guidelines, optimizing care for individuals with acute respiratory failure.

The generalizability of these findings is shaped by factors such as study populations, clinical settings, and the interventions assessed. The included trials involved diverse patient groups with acute respiratory failure, including those with COPD exacerbations, cardiogenic pulmonary edema, and hypoxemic respiratory failure. While the findings are broadly applicable across various clinical scenarios, the heterogeneity in study populations, settings, and interventions highlights the need for further research to refine the indications and optimize the use of NIV for preoxygenation in intubation.

### Strengths and limitations

This study has several strengths, including frequentist and Bayesian approaches, providing a robust and nuanced interpretation of the data by considering statistical significance and probabilistic effect sizes. The inclusion of diverse patient populations, such as those with COPD exacerbations, cardiogenic pulmonary edema, and hypoxemic respiratory failure, enhances the generalizability of the findings. Rigorous data collection protocols ensured high-quality data and minimized bias. However, limitations remain, including potential selection and publication biases, relatively small sample sizes in some studies affecting the power and generalizability, and significant heterogeneity across patient populations, settings, and interventions, complicating pooled result interpretation. While random-effects models account for between-study variability, publication bias remains a concern. This study incorporated recent studies and advanced statistical methods, offering a more comprehensive evaluation of NIV *versus* BVMV in preoxygenation compared to previous meta-analyses. Future research should address these limitations to refine the evidence base and optimize clinical recommendations.

## CONCLUSION

Our study demonstrates that noninvasive ventilation significantly reduces the risk of hypoxemia compared to bag-valve-mask ventilation during preoxygenation for orotracheal intubation. However, noninvasive ventilation showed no significant impact on secondary outcomes like all-cause mortality, regurgitation, ventilation time, or intensive care unit stay, highlighting the need for careful patient selection. Future research should identify subgroups most likely to benefit from noninvasive ventilation and further assess its safety concerning regurgitation and aspiration, to optimize its clinical application and refine guidelines.

## Data Availability

The contents underlying the research text are included in the manuscript
