# Supplementary material for: Effectiveness of noninvasive ventilation for preoxygenation in emergency intubation: a systematic review and meta-analysis
Source: Crit Care Sci. 2026 Jan 14;38:e20260128. doi: 10.62675/2965-2774.20260128 (PMC12977216; doi:10.62675/2965-2774.20260128)
Supplement: Supplementary file 1 [file 2965-2774-ccsci-38-e20260128-suppl1.pdf]

Critical Care Science
https://doi.org/10.62675/2965-2774.20260128

SUPPLEMENTARY MATERIAL

Effectiveness of noninvasive ventilation for preoxygenation in emergency intubation: a systematic review and meta-analysis

Luciana Gioli-Pereira[1,2], Victor A Gomez Galeano[3], Rafael Hortencio Melo[1], Camila Campos Grisa Padovese[4], Edielle Sant'Anna Melo[2], Ary Serpa Neto[1,5]

Table 1S - Search strategy

Search strategy for each database	
PubMed	("critically ill adults" OR "critical illness" OR "ICU patients") AND ("tracheal intubation" OR "endotracheal intubation") AND ("preoxygenation" OR "noninvasive ventilation" OR "oxygen mask") AND (randomized controlled trial[pt] OR controlled clinical trial[pt] OR clinical trials as topic[mesh:noexp] OR trial[ti] OR random*[tiab] OR placebo*[tiab])
EMBASE	('critically ill adults' OR 'critical illness' OR 'ICU patients') AND ('tracheal intubation' OR 'endotracheal intubation') AND ('preoxygenation' OR 'noninvasive ventilation' OR 'oxygen mask') AND ('controlled clinical trial'/exp OR (random* OR placebo*):ti,ab OR trial:ti) AND [embase]/lim
Cochrane Library	("critically ill adults" OR "critical illness" OR "ICU patients") AND ("tracheal intubation" OR "endotracheal intubation") AND ("preoxygenation with noninvasive ventilation" OR "noninvasive ventilation" OR "preoxygenation with oxygen mask")

Table 2S - Outcomes definitions

Outcome	Definition
Hypoxemia	Hypoxemia during intubation was defined by an oxygen saturation of less than 85% during the interval between induction of anesthesia and 2 minutes after tracheal intubation
All-cause mortality	Death for any cause in the ICU within 28 days
ICU LOS	Duration of stay in the ICU
Ventilation time	Duration of ventilation time
Regurgitation	Presence of gastric content seen during laryngoscopy

ICU - intensive care unit; LOS - length of stay.

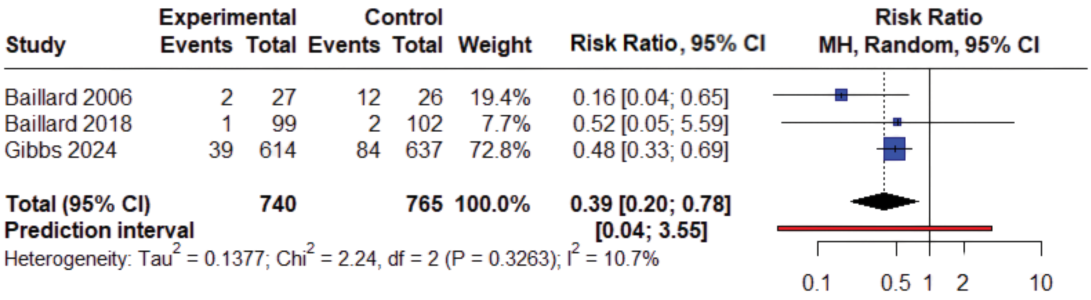

Figure 1S - Forest plot of frequentist analysis of hypoxemia

Effect sizes left of 1 (or left of 0 in log scale) favor the experimental group (noninvasive ventilation), while those right of 1 (or 0 in log scale) favor the control group (bag-valve mask ventilation).

This is an open access article under the CC BY license (https://creativecommons.org/licenses/by/4.0/).

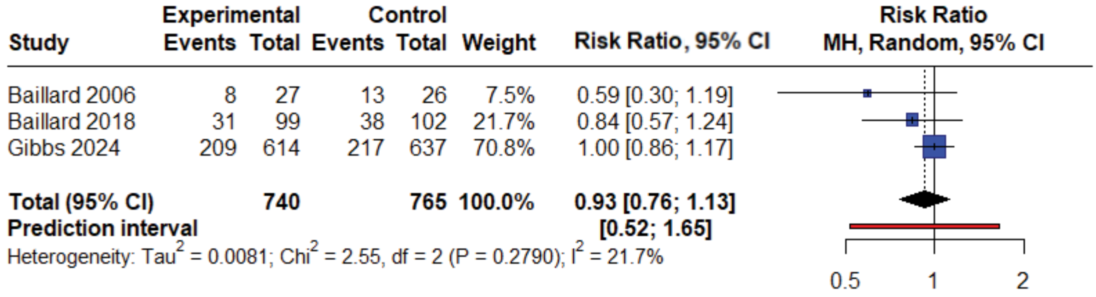

Figure 2S - Forest plot of frequentist analysis of mortality.

Effect sizes left of 1 (or left of 0 in log scale) favor the experimental group (noninvasive ventilation), while those right of 1 (or 0 in log scale) favor the control group (bag-valve mask ventilation).

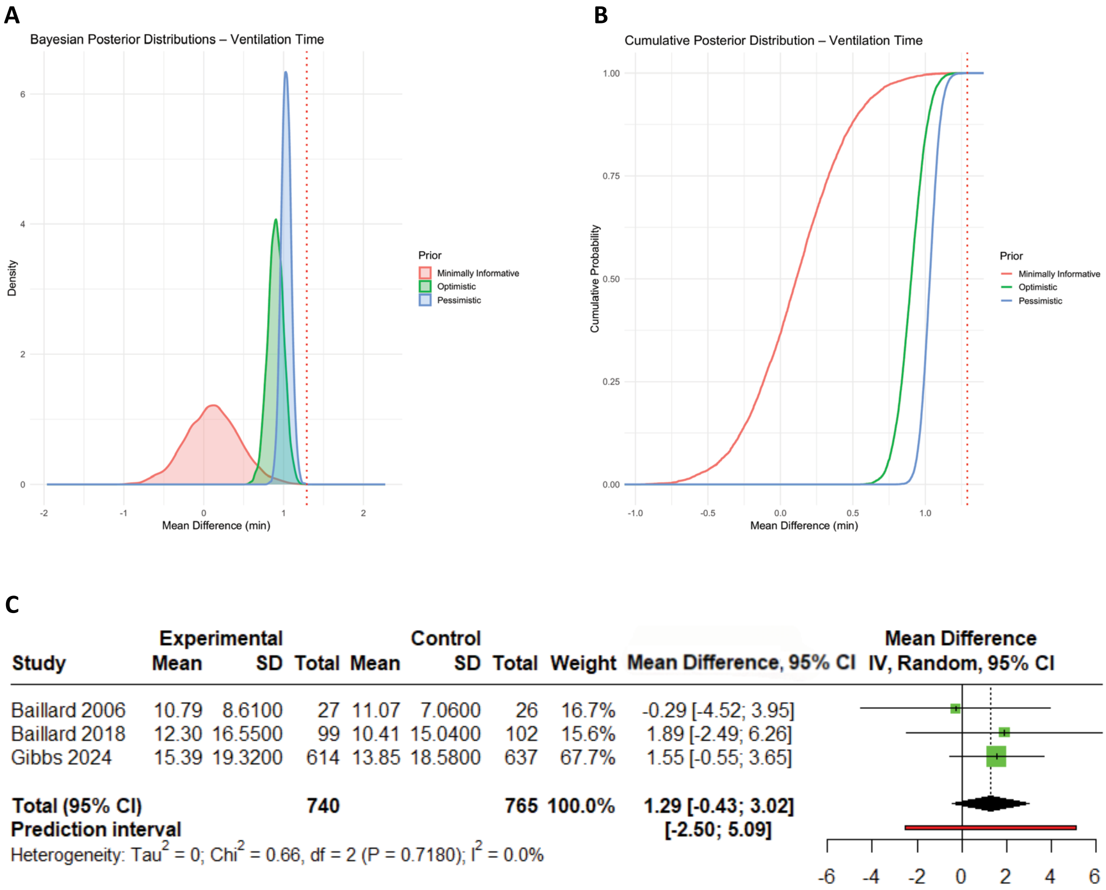

Figure 3S - Bayesian models for ventilation time.

A) Bayesian model; B) cumulative posterior distribution; C) forest plot of frequentist analysis for ventilation time.

For continuous outcomes, negative values favor the experimental group (noninvasive ventilation) if the outcome is reduced (e.g., ventilation time), while positive values favor the experimental group (noninvasive ventilation) if the outcome is increased (e.g., oxygenation).

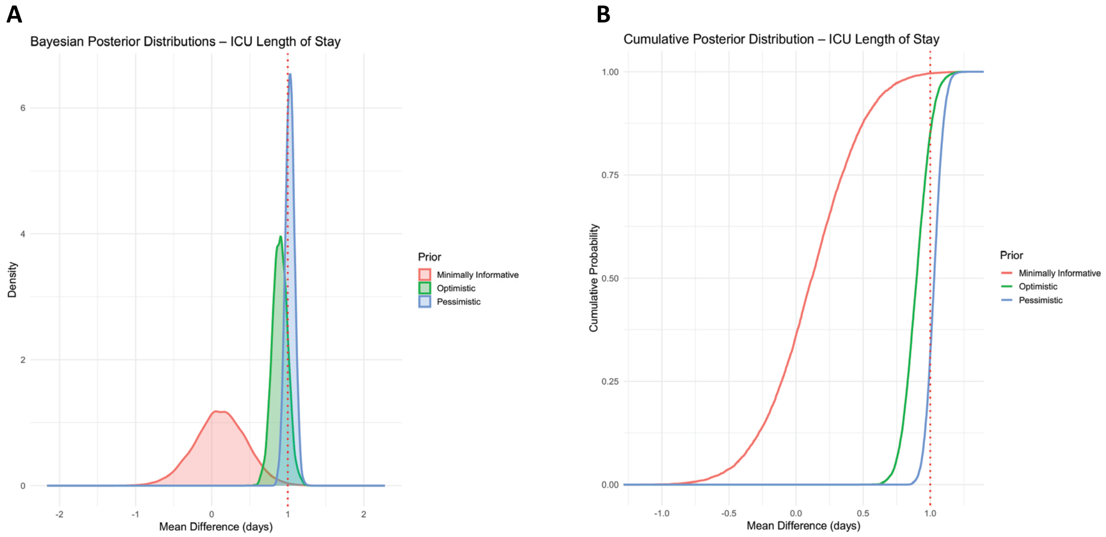

C

Study	Experimental Mean	Experimental SD	Experimental Total	Control Mean	Control SD	Control Total	Weight	Mean Difference, 95% CI
Baillard 2006	18.36	11.7400	27	17.36	8.6280	26	7.8%	1.00 [-4.53; 6.53]
Baillard 2018	7.17	12.7900	99	5.11	9.0240	102	25.4%	2.06 [-1.01; 5.12]
Gibbs 2024	12.85	17.0910	614	12.25	17.0900	637	66.7%	0.60 [-1.30; 2.49]
**Total (95% CI)**			**740**			**765**	**100.0%**	**1.00 [-0.55; 2.55]**
**Prediction interval**								**[-2.40; 4.40]**

Heterogeneity: $Tau^2 = 0$; $Chi^2 = 0.63$, df = 2 (P = 0.7306); $I^2 = 0.0\%$

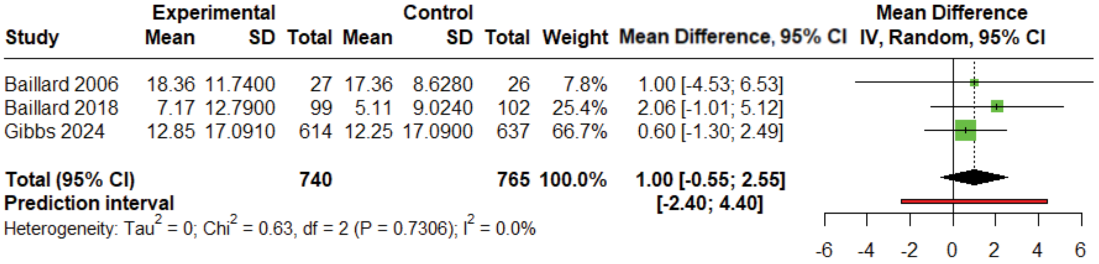

Figure 4S - Length of stay in intensive care unit.

A) Bayesian model; B) cumulative posterior distribution; C) forest plot of frequentist analysis for length of stay in intensive care unit.

For continuous outcomes, negative values favor the experimental group (noninvasive ventilation) if the outcome is reduced, while positive values favor the experimental group (noninvasive vantilation) if the outcome is increased.

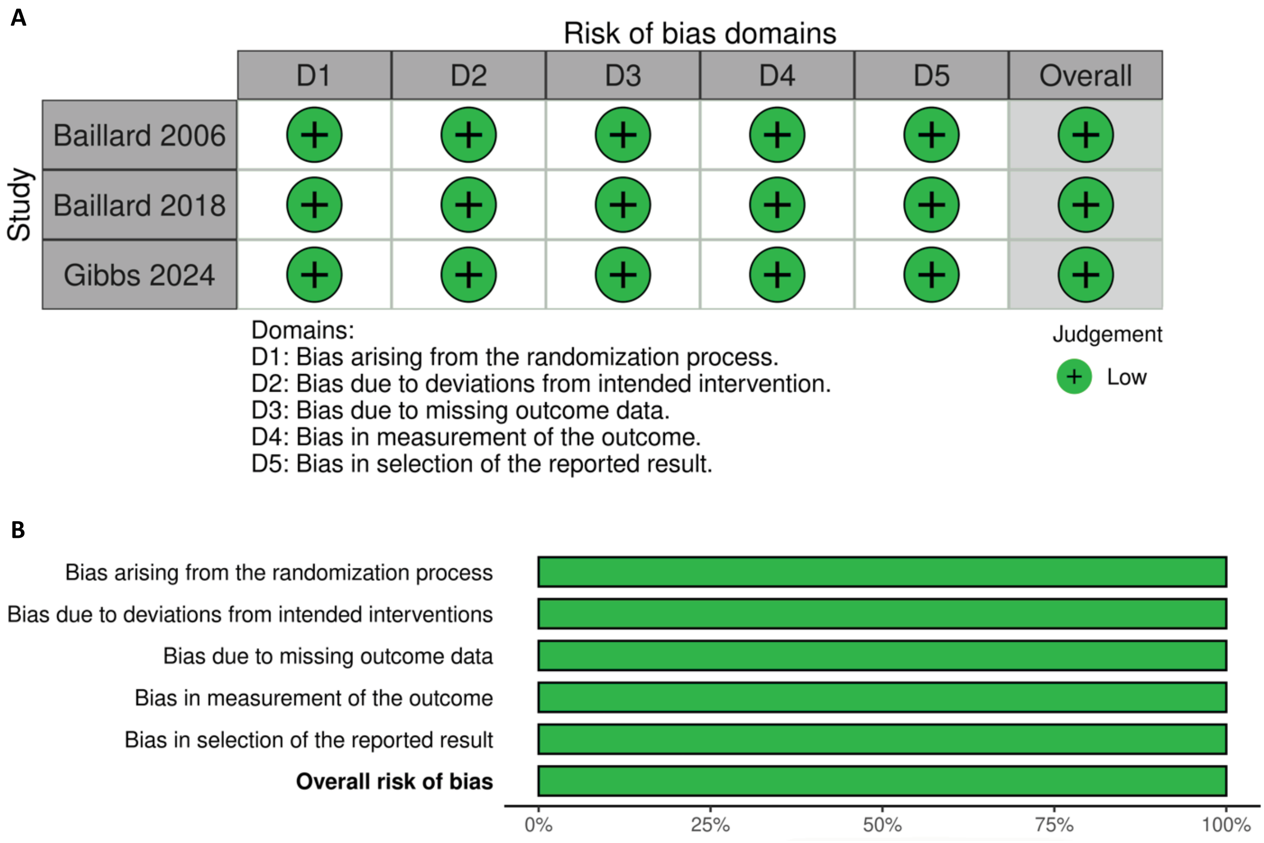

Figure 5S - Risk of bias 2 (RoB-2) of all included studies.
A) "Traffic light" plot of the domain-level judgments for each study; B) summary of overall weighted bar plot of risk-of-bias judgments within each bias domain.
